# Draft genome sequence of *Gordonia* sp. ABKF26, a potential petroleum, plastic, and rubber-degrading bacterium, isolated from St. Albans Bay, VT, USA

**DOI:** 10.1128/mra.00876-24

**Published:** 2024-09-30

**Authors:** Kermisha Felix, Ashley Barkley, Luke Myers, Nana Y. D. Ankrah

**Affiliations:** 1 Biological Sciences Department, State University of New York at Plattsburgh, Plattsburgh, New York, USA; 2 Lake Champlain Research Institute, State University of New York at Plattsburgh, Plattsburgh, New York, USA; Rochester Institute of Technology, Rochester, New York, USA

**Keywords:** *Gordonia*, Lake Champlain, bioremediation, petroleum degradation, plastic degradation, rubber degradation

## Abstract

Here, we present the draft genome sequence of *Gordonia* sp. ABKF26, a potential petroleum, plastic, and rubber degrading bacterium isolated from Lake Champlain. The assembled genome comprises a 6.0-Mb chromosome with a GC content of 67.8%. *Gordonia* sp. ABKF26 is predicted to be lysogenized by one intact 52.2 Kbp prophage.

## ANNOUNCEMENT

The actinomycete genus *Gordonia* includes members of biotechnological, ecological, and medical relevance (1, 2). For example, several *Gordonia* species have been shown to degrade aromatic hydrocarbons found in petroleum (3), plastic (phthalate) (4), and rubber (polyisoprene) (5). Members of the genus *Gordonia* are also important algal symbionts (6), and some are opportunistic pathogens that can cause disease in humans (7).

In this study, *Gordonia* sp. ABKF26 was isolated from St. Albans Bay, VT, USA (44.786667 N 73.170270 W) in September 2022, as previously described (8). Briefly, lake water was collected at 3 m depth, inoculated on tryptic soy agar plates (Hardy Diagnostics), and incubated for 7 days at 25°C in the dark. Isolated colonies were purified by three rounds of re-streaking to generate pure cultures.

Genomic DNA (gDNA) was extracted from a 48-hour-old culture generated by inoculating a single bacterial colony into tryptic soy broth (Hardy Diagnostics) and incubating at 25°C without shaking. The gDNA was extracted using the ZymoBIOMICS DNA/RNA Miniprep Kit (Zymo Research). Library preparation, sequencing, and genome assembly were performed by Plasmidsaurus (Eugene, OR, USA). Sequencing libraries were prepared using the Rapid Barcoding Sequencing Kit version SQK-RBK114.96 (Oxford Nanopore Technologies) according to the manufacturer’s instructions with no fragmentation or size selection of DNA. Sequencing was conducted on the PromethION P24 platform (Oxford Nanopore Technologies), equipped with R.10.4.1 flow cells and base calling performed using Dorado v7.1.4 on the super-accurate mode (Oxford Nanopore Technologies).

Default parameters were used for all software, unless otherwise specified. Reads were filtered with Filtlong v0.2.1 (https://github.com/rrwick/Filtlong). Reads were quality-filtered using a minimum Phred score of 15 and minimum length of 1,000  bp. The genome was assembled and polished using Flye v2.9.1 (9) and Medaka v1.8.0 (https://github.com/nanoporetech/medaka), respectively. Assembly completeness and contamination were assessed using CheckM v1.2.2 (10). Genome annotation was performed using the NCBI Prokaryotic Genome Annotation Pipeline v 6.7 (11) and RAST v2.0 (12). A summary of assembly statistics and genome overview is provided in Table 1.

**TABLE 1:** Summary of assembly statistics and genome overview

Genome coverage	213 x
CheckM completeness	99.82
CheckM contamination	0.36
Number of contigs	8
Total bp sequenced	1,271,535,865 bp (1272 Mb)
Total number of reads	603,448
Longest read (bp)	47,082
Read N50	3,298
Contig N50	5,631,740
Genome size (bp)	5,955,660
GC content (%) of the genome	67.8%
Genes (total)	5,509
CDSs (total)	5,451
Genes (coding)	5,377
rRNAs	3, 3, and 3 (5S, 16S, and 23S)
tRNAs	46
ncRNAs	3

The genome sequence of *Gordonia* sp. ABKF26 is 5,955,660 bp (6.0 Mb) with a GC content of 67.8%. The genome contains 5,377 protein coding genes and 46 tRNAs (Table 1). The genome of *Gordonia* sp. ABKF26 encodes several protein-coding genes predicted to be involved in terpenoid and aromatic hydrocarbon degradation including latex-clearing protein, phthalate dioxygenase reductase, benzoate dioxygenase, and toluene hydroxylase. *Gordonia* sp. ABKF26 also encodes many protein-coding genes predicted to be involved in the synthesis of antimicrobial compounds. The antiSMASH v7.1.0 software (13) analysis predicted 15 putative secondary metabolite biosynthetic gene clusters including regions with genes coding for ectoine and ε-poly-L-lysine. *Gordonia* sp. ABKF26 is predicted to be lysogenized by one intact prophage. The prophage has a genome size of 52.2 Kbp and 64 predicted phage genes. The intact prophage was identified using PHASTEST v3.0 (14).

## Data Availability

The draft genome sequence of *Gordonia* sp. ABKF26 has been deposited at DDBJ/ENA/GenBank under the accession no. JBGCVZ000000000. The version described in this paper is version JBGCVZ010000000. The associated BioProject and BioSample accession numbers are PRJNA1139760 and SAMN42780774, respectively. Raw sequence reads have been submitted to the Sequence Read Archive (accession no.: SRR30071187).

## References

[1] Arenskötter M, Bröker D, Steinbüchel A. 2004. Biology of the metabolically diverse genus Gordonia. Appl Environ Microbiol 70:3195–3204. doi:10.1128/AEM.70.6.3195-3204.2004 15184112 PMC427784

[2] Sowani H, Kulkarni M, Zinjarde S. 2018. An insight into the ecology, diversity and adaptations of Gordonia species. Crit Rev Microbiol 44:393–413. doi:10.1080/1040841X.2017.1418286 29276839

[3] Yang Y, Zhang W, Zhang Z, Yang T, Xu Z, Zhang C, Guo B, Lu W. 2023. Efficient bioremediation of petroleum-contaminated soil by immobilized bacterial agent of Gordonia alkanivorans W33. Bioeng (Basel) 10:561. doi:10.3390/bioengineering10050561 PMC10215891 37237630

[4] Zhang H, Lin Z, Liu B, Wang G, Weng L, Zhou J, Hu H, He H, Huang Y, Chen J, Ruth N, Li C, Ren L. 2020. Bioremediation of di-(2-ethylhexyl) phthalate contaminated red soil by Gordonia terrae RL-JC02: characterization, metabolic pathway and kinetics. Sci Total Environ 733:139138. doi:10.1016/j.scitotenv.2020.139138 32446058

[5] Sarkar B, Mandal S. 2021. Gordonia sp. BSTG01 isolated from Hevea brasiliensis plantation efficiently degrades polyisoprene (rubber). 3 Biotech 11:508. doi:10.1007/s13205-021-03063-5 PMC8607378 34881168

[6] Liu W, Xing X, Dong Q, Liu X, Li W. 2024. Isolation and identification of the alga-symbiotic bacterium Gordonia and characterisation of its exopolysaccharide. Nat Prod Res 38:523–529. doi:10.1080/14786419.2022.2123477 36102747

[7] Sowani H, Kulkarni M, Zinjarde S, Javdekar V. 2017. *Gordonia* and related genera as opportunistic human pathogens causing infections of skin, soft tissues, and bones, p 105–121. In The microbiology of skin, soft tissue, bone and joint infections. Elsevier.

[8] Barkley A, Felix K, Myers L, Ankrah NYD. 2024. Genome sequence of Bacillus pumilus RI06-95 isolated during a Microcystis bloom in lake Champlain, USA. Microbiol Resour Announc. doi: 10.1128/mra.00713-24 10.1128/mra.00713-24 PMC11465950 39212350

[9] Kolmogorov M, Yuan J, Lin Y, Pevzner PA. 2019. Assembly of long, error-prone reads using repeat graphs. Nat Biotechnol 37:540–546. doi:10.1038/s41587-019-0072-8 30936562

[10] Parks DH, Imelfort M, Skennerton CT, Hugenholtz P, Tyson GW. 2015. CheckM: assessing the quality of microbial genomes recovered from isolates, single cells, and metagenomes. Genome Res 25:1043–1055. doi:10.1101/gr.186072.114 25977477 PMC4484387

[11] Tatusova T, DiCuccio M, Badretdin A, Chetvernin V, Nawrocki EP, Zaslavsky L, Lomsadze A, Pruitt KD, Borodovsky M, Ostell J. 2016. NCBI prokaryotic genome annotation pipeline. Nucleic Acids Res 44:6614–6624. doi:10.1093/nar/gkw569 27342282 PMC5001611

[12] Aziz RK, Bartels D, Best AA, DeJongh M, Disz T, Edwards RA, Formsma K, Gerdes S, Glass EM, Kubal M, et al.. 2008. The RAST server: rapid annotations using subsystems technology. BMC Genomics 9:75. doi:10.1186/1471-2164-9-75 18261238 PMC2265698

[13] Blin K, Shaw S, Augustijn HE, Reitz ZL, Biermann F, Alanjary M, Fetter A, Terlouw BR, Metcalf WW, Helfrich EJN, van Wezel GP, Medema MH, Weber T. 2023. antiSMASH 7.0: new and improved predictions for detection, regulation, chemical structures and visualisation. Nucleic Acids Res 51:W46–W50. doi:10.1093/nar/gkad344 37140036 PMC10320115

[14] Wishart DS, Han S, Saha S, Oler E, Peters H, Grant JR, Stothard P, Gautam V. 2023. PHASTEST: faster than PHASTER, better than PHAST. Nucleic Acids Res 51:W443–W450. doi:10.1093/nar/gkad382 37194694 PMC10320120

